# Embryonic stem cell-derived extracellular vesicles enhance the therapeutic effect of mesenchymal stem cells

**DOI:** 10.7150/thno.35305

**Published:** 2019-09-21

**Authors:** Yan Zhang, Jia Xu, Siying Liu, Meikuang Lim, Shuang Zhao, Kaige Cui, Kaiyue Zhang, Lingling Wang, Qian Ji, Zhongchao Han, Deling Kong, Zongjin Li, Na Liu

**Affiliations:** 1 School of Medicine, Nankai University, Tianjin, 300071, China; 2 Key Laboratory of Bioactive Materials, Ministry of Education, College of Life Sciences Nankai University, Tianjin, 300071, China; 3 College of Life Sciences, Nankai University, Tianjin, 300071, China; 4 Department of Radiology, Tianjin First Central Hospital, Tianjin, China; 24 Fukang Road, Nankai District, Tianjin, 300071, China; 5 Beijing Engineering Laboratory of Perinatal Stem Cells, Beijing Institute of Health and Stem Cells, Health & Biotech Co., Beijing, 100176, China

**Keywords:** Cellular senescence, Mesenchymal stem cells, Embryonic stem cells, Extracellular vesicles, IGF1/PI3K/AKT pathway

## Abstract

**Background:** Embryonic stem cells (ES) have a great potential for cell-based therapies in a regenerative medicine. However, the ethical and safety issues limit its clinical application. ES-derived extracellular vesicles (ES-EVs) have been reported suppress cellular senescence. Mesenchymal stem cells (MSCs) are widely used for clinical cell therapy. In this study, we investigated the beneficial effects of ES-EVs on aging MSCs to further enhancing their therapeutic effects.

**Methods:** *In vitro*, we explored the rejuvenating effects of ES-EVs on senescent MSCs by senescence-associated β-gal (SA-β-gal) staining, immunostaining, and DNA damage foci analysis. The therapeutic effect of senescent MSC pre-treated with ES-EVs was also evaluated by using mouse cutaneous wound model.

**Results:** We found that ES-EVs significantly rejuvenated the senescent MSCs *in vitro* and improve the therapeutic effects of MSCs in a mouse cutaneous wound model. In addition, we also identified that the IGF1/PI3K/AKT pathway mediated the antisenescence effects of ES-EVs on MSCs.

**Conclusions:** Our results suggested that ES cells derived-extracellular vesicles possess the antisenescence properties, which significantly rejuvenate the senescent MSCs and enhance the therapeutic effects of MSCs. This strategy might emerge as a novel therapeutic strategy for MSCs clinical application.

## Introduction

Mesenchymal stem cells (MSCs), derived from several kinds of tissues such as placenta, umbilical cord, bone marrow and adipose tissue, are multipotent stem cells that can differentiate into many cell types. MSCs have been recognized as important candidates for the treatment of many degenerative diseases or injuries, ranging from ischemic diseases and renal failure to cutaneous injury 1-3. Furthermore, MSCs can be expanded by continuously passage *in vitro*, to obtain a sufficient number of cells that can be used for clinical applications. Along with the continuous passage *in vitro*, MSCs exhibit the senescence-associated features, including enlarged morphology, irreversible growth arrest, enhanced SA-β-gal activity, decreased stemness of stem cells, increased cell apoptosis and DNA damage foci, and telomere attrition 4, 5. For senescence MSCs, the characteristics of stem cell are lost, and their therapeutic effects are limited. Excessive and aberrant accumulation of senescent cells in tissues negatively affects regenerative capacities and accelerates the progress of various age-related diseases, including cancer 6-8.

Therefore, researchers attempt to find a better way to block the cellular senescence. One study found the melatonin could protect MSCs from hydrogen peroxide (H_2_O_2_) induced premature senescence via the silent information regulator type 1 (SIRT1)-dependent pathway 9. Vitamin C also exert efficient rescue for many feature in premature senescent MSCs and restore the viability of MSCs in mice cutaneous wound model 10. These studies provide a novel type of treatment of cellular senescence and some age-related diseases.

Mouse ES cells, derived from the blastocyst stage embryos, are distinguished by their ability to self-renew and differentiate into all cell types 11. Because of their plasticity and potentially unlimited capacity of self-renewal, embryonic stem cells have been proposed for regenerative medicine and tissue replacement after injury or diseases. The major barriers to the possible transplantation of ES cells into patients are immune rejection and the risk of forming tumors 12. It has been reported that conditioned medium from ES cells (ES-CM) has beneficial effects on cell proliferation and tissue regeneration via the factors secreted from ES cells 13. Recently studies suggested that extracellular vesicles (EVs), which are biological particles released by many cell types, could be considered for therapeutic utility 14, 15. The EVs transfer proteins and nucleic acids between cells and play an important role in the target cells. Moreover, EVs isolated from various types of stem cells have different properties such as anti-apoptosis, pro-angiogenesis, and anti-fibrosis 14, 16-18. Recently, one study showed that mmu-miR-291a-3p derived from the ES-CM inhibited the cellular senescence in human dermal fibroblasts through the TGF-β receptor 2 pathway 19.

In this study, we explored the effects of EVs derived from ES cells (ES-EVs) on the senescent MSCs. Our results indicated that ES-EVs rejuvenated the senescent MSCs and enhanced their therapeutic effects *in vivo*. Furthermore, we found that the IGF1/PI3K/AKT pathway mediated the antisenescence activities of ES-EVs on MSCs.

## Methods

### Cell culture

The mouse D3 ES cell lines were grown on plates pre-coated with 0.1% gelatin and cultured in DMEM medium (Corning) supplemented with 15% FBS (Hyclone), 1% L-Glutamine (Corning), 1% NEAA (Gibco), 1% penicillin/streptomycin (Gibco), 1% β-mercaptoethanol (Sigma), and 1000 units/mL of LIF (Millipore) at 37 °C in a 5% CO_2_ incubator. The MSCs were isolated as described previously 20 and cultured in Dulbecco's Modified Eagle's Medium (DMEM)/ F12 medium (Gibco) with 10% bovine fetal bovine serum (FBS; HyClone) and 100 U/mL penicillin-streptomycin (Gibco).

### Collection of conditioned medium

ES cells were cultured in ES cells medium until reaching 70% confluence at 37 °C in a 5% CO_2_ incubator. ES cells were washed 3 times with PBS then incubated in DMEM/F12 (Gibco) at 37 °C for 24 h. ES cells derived conditioned medium (ES-CM) was collected, centrifuged for 15 min at 500 g. MSCs were cultured in MSCs medium until reaching 70% confluence at 37 °C in a 5% CO_2_ incubator. MSCs were washed 3 times with PBS then incubated in DMEM/F12 (Gibco) at 37 °C for 24 h. MSCs-derived conditioned medium (MSC-CM) was collected, centrifuged for 15 min at 500 g.

### Establishment of senescent cell model

To establish the senescent cell model, the MSCs were sub-cultured serially for 18 passages. The senescent MSCs have higher level of SA-β-gal and lower proliferation ability *in vitro*.

### Extracellular vesicles isolation

Extracellular vesicles were purified from supernatants of ES cells by differential centrifugations as previously described 21. In brief, ES cells were cultured for 24 h in DMEM/F12 medium. The cell culture medium was collected by centrifuging at 500 g for 10 min to remove any cell contaminations. Then the cell debris and apoptotic bodies were discarded by a centrifugation step of 2000 g for 30 min. ES-EVs were isolated by ultracentrifugation at 100000 g for 30 min at 4 °C. Finally, the extracellular vesicles were isolated by a second ultracentrifugation at 100000 g for 2 h at 4 °C.

### Extracellular vesicles characterization

The particle size of the final EVs pellets were determined by dynamic light scattering measurements using a BI-200SM laser scattering instrument (ZetaPALS, Brookhaven, NY) at 20 °C. The morphology of the EVs was verified by transmission electron microscopy (TEM; TalosF200C, Hillsboro, OR). A drop of EVs pellets (20 μL) were absorbed by a film (Zhongjingkeji Technology, Beijing, China) and the phosphotungstic acid was used for negative staining, and samples were air-dried for image capturing by TEM. A BCA Protein Assay Kit (Promega, Madison, WI) was used to measure the protein concentration in EVs.

### Extracellular vesicles internalization

EVs were labeled with the CM-Dil membrane dye (Invitrogen, Carlsbad, CA) following the manufacturer's protocol. Briefly, EVs were mixed with 1 μmol/L CM-Dil, and incubated for 5 min at room temperature. Excess dye was removed by ultracentrifugation at 100,000 g for 70 min at 4 °C, and the pellets were washed three times. The final labeled EVs were re-suspended in PBS. Labeled EVs were co-cultured with MSCs expressing green fluorescent protein (GFP). MSCs were washed with PBS and fixed in 4% paraformaldehyde. The uptake of EVs was observed by fluorescence microscopy.

### Cell proliferation assay

MSCs (3×10^3^ cells/well) were seeded on 96-well plates with FBS-free medium in the presence of ES-CM for 12, 24 and 48 h, respectively. Thiazolyl blue tetrazolium bromide (MTT) solution (Sigma) was added into each well and incubated for 4 h at 37 °C. The supernatant was then removed and the formazan crystals were dissolved in the dimethyl sulfoxide. The optical density was measured at 490 nm using a microplate reader (Promega). As for the experiment of Ki-67 staining, MSCs were treated with basal DMEM/F12, MSC-CM and ES-CM for 48 h, respectively. Then, the cells on the cover slips were subjected to immunofluorescence staining (Rabbit anti-Ki-67, Abcam). Photographs of three random fields of view were imaged with fluorescence microscope (Nikon, Tokyo, Japan). The quantification of Ki-67^+^ cells was analyzed by using Image J software.

### RNA isolation and Real-time PCR analysis

Total RNA was extracted from the cells with 500 μL TRIzol (Invitrogen, Grand Island, NY) according to instructions supplied by the manufacturer. Subsequently, cDNA was synthesized from RNA using the BioScript All-in-One cDNA Synthesis SuperMix (Bimake, Houston, TX) and real-time PCR was performed with the Opticon® System (Bio-Rad, Hercules, CA) using Hieff™ qPCR SYBR® Green Master Mix (No Rox) (Yeasen). Relative gene expression folding changes were identified with the 2^-ΔΔCt^ method. The sequences of primers used in this study are shown in **Table S1.**

### Western blotting analysis

Cells were harvested in RIPA lysis buffer (Solarbio, Shanghai, China), quantified by a BCA Protein Assay Kit (Promega), separated by 10% SDS-PAGE and transferred to polyvinylidene fluoride membranes (Millipore, Darmstadt, Germany). After blocking with 5% skim milk for 2 h, the membranes were incubated with primary antibodies at 4 °C overnight. After three times washing with TBST, the membranes were incubated with secondary antibodies for 2 h at room temperature. The Pierce enhanced chemiluminescence western blotting substrate (Millipore) was used to detect the signal. The primary antibodies were used for western blot analysis: rabbit anti-Sox2 (Santa Cruz Biotechnology), rabbit anti-Oct4 (Santa Cruz Biotechnology), rabbit anti-Nanog (Bethyl), rabbit anti-P53 (Santa Cruz Biotechnology), rabbit anti-P16 (Wanleibio, Shenyang, China), rabbit anti-CD9 (Abcam, Cambridge, UK), rabbit anti-CD63 (Wanleibio, Shenyang, China), rabbit anti-IGF1R (Novus biological), mouse ant-IGF1 (Novus biological).

### Immunofluorescence microscopy

Cells were fixed with 4% formaldehyde in PBS at room temperature (RT) for 10 min. After fixation, cells were treated with 0.1% Triton X-100 in PBS for 10 min at RT. After blocked with 10% BSA for 2 h, cells were incubated with the primary antibody at 4 °C overnight, followed by washing in PBS for three times and incubation at RT for 2 h with the corresponding secondary antibody. The following antibodies were used at the indicated dilutions: anti-Ki67 (Abcam), anti-γ-H2AX (Cell signaling technology).

### Senescence associated β-galactosidase (SA-β-gal) staining

SA-β-gal staining was performed as described previously 22. Briefly, cultured cells were washed with PBS and fixed in staining fixatives for 15 min at room temperature. Fixed cells were stained with fresh SA-β-gal staining solution 37 °C overnight (Beyotime Biotechnology, China).

### Cell apoptosis assay

The activity of Caspase-3 was measured with Caspase-3 colorimetric assay kit (Beyotime Biotechnology, China) according to the manufacturer's instruction. Briefly, cells were harvested after treatment using lysis buffer containing DTT. Each sample (200 mg per sample) was incubated with 2 x reaction buffer and probe for Caspase-3 at 37 °C for 4 h. The optical density was measured at 400 nm using a microplate reader (Promega).

### Flow cytometry analysis

The cells were mixed with the pre-cooled PBS, and the procedure was repeated 3 times followed by resuspension of cells to prepare the cell suspension at a density of 5×10^5^ cells/mL, in which we extracted 1 mL suspension for centrifugation and the supernatant was discarded. In the sediment, 500 µL binding buffer, 5 µL Annexin V-FITC (Cwbio) and 10 µL propidium iodide (PI) were sequentially added. The suspension was then incubated in the dark for 10 min. A flow cytometer (Thermo Fisher Scientific, Inc, Waltham, MA, USA) was used to detect cell apoptosis in the ES-CM and negative control group.

### In vivo wound-healing assay

BALB/c mice were used to establish cutaneous wounding model. A punch wound was created at the dorsal surface exactly between the cervical root and shoulder of each mouse as previously published 23, Mice were individually anesthetized using an intraperitoneal injection of chloral hydrate (330 mg/kg), then shaved the hair of the dorsal surface with an electric clipper. A full-thickness wound (approximately 10 mm in diameter) was created by excising the skin and the underlying panniculus carnosus. Wounds were circumscribed by donut-shaped silicone splints (internal diameter: 10 mm, external diameter: 15 mm) held in place using 6-0 nylon sutures to prevent wound contraction. Mice were injected with 5×10^5^ MSCs/100 μL PBS (n=10 for each group) into the injured area.

### Bioluminescence imaging (BLI) analysis

For real-time monitoring of the cell fate of MSCs *in vivo*, BLI was performed using the Imaging System IVIS Lumina (Xenogen Corporation, Hopkinto, MA) as reported previously 24. Peak BLI signal was quantified by average radiance from a fixed-area region of interest (ROI) over skin wound area.

### Histological analysis

Hematoxylin Eosin (HE) staining and Masson's staining were performed to investigate the effects of MSC treatment at day 12 (n = 5 per group). For quantification of the collagen deposition in skin wound areas, microscopic fields of Masson's staining were measured using Image J software. The quantification for collagen deposition was expressed as the average percentage of collagen contents in the field of view.

### Statistical analysis

Quantitative data were presented as the means±SEM of 3 independent experiments for each condition. Statistical analysis was performed by one- or two-way ANOVA using GraphPad (GraphPad Prism Software Inc., San Diego, CA, USA). Statistical significance was indicated at P < 0.05.

## Results

### Cellular senescence in MSCs after continuous passages

Mesenchymal stem cells can be widely used in the clinical treatment of various diseases, but before the application, due to the limitation of the number of cells, it is often necessary to expand the cells. In order to understand the changes in cellular senescence after multiple passages of MSCs, MSCs were continuously passaged for 18 passages *in vitro*. We used the passage 6 (P6) and passage 18 (P18) MSCs to detect the senescence-associated features. We found that P18 MSCs exhibited morphologically characterized aging, such as the loss of long fusiform morphology, which in turn becomes more broad and flat compared to P6 MSCs **(Figure S1A)**. We also found the proliferation of P18 MSCs was decreased compared to P6 MSCs by MTT assay **(Figure S1B)**. To further investigate the proliferative capability of late-passaged MSCs, we employed Ki67 (a proliferation marker) staining and found the percentage of Ki67 positive cells significantly decreased in the P18 MSCs **(Figure S1C)**, which suggesting that the proliferation potential decreased in late-passage of MSCs.

To verify the cellular senescence of later-passaged MSCs, we employed senescence associated β-galactosidase (SA-β-gal) staining. The results showed that the percent of senescent MSCs that stained positive for SA-β-gal were markedly higher in P18 MSCs **(Figure S2A)**. The number of nuclear foci for phosphorylated ATM/ATR substrates γ-H2AX was also increased in P18 MSCs** (Figure S2B)**. Compared with P6 MSCs, more cells harbored more than 5 foci of γ-H2AX in P18 MSCs. In parallel, several senescence-associated genes were also significantly increased in P18 MSCs, such as P16, P21, P53, GADD45B, and interleukin-6 (IL-6) **(Figure S2C).** The increased expression of P53 and P16 were also confirmed by western blot analysis **(Figure S2D)**. Simultaneously, the expression of stemness related genes (OCT4, SOX2, NANOG, and KLF4) decreased in P18 MSCs **(Figure S1D)**. Taken together, all of the results above indicated that some senescence-associated features occur in late-passaged MSCs, including cell cycle arrest, increased SA-β-gal activity, increased DNA damage and reduced stemness.

### Effect of ES-CM on the proliferation ability of senescent MSCs

**Figure 1:**
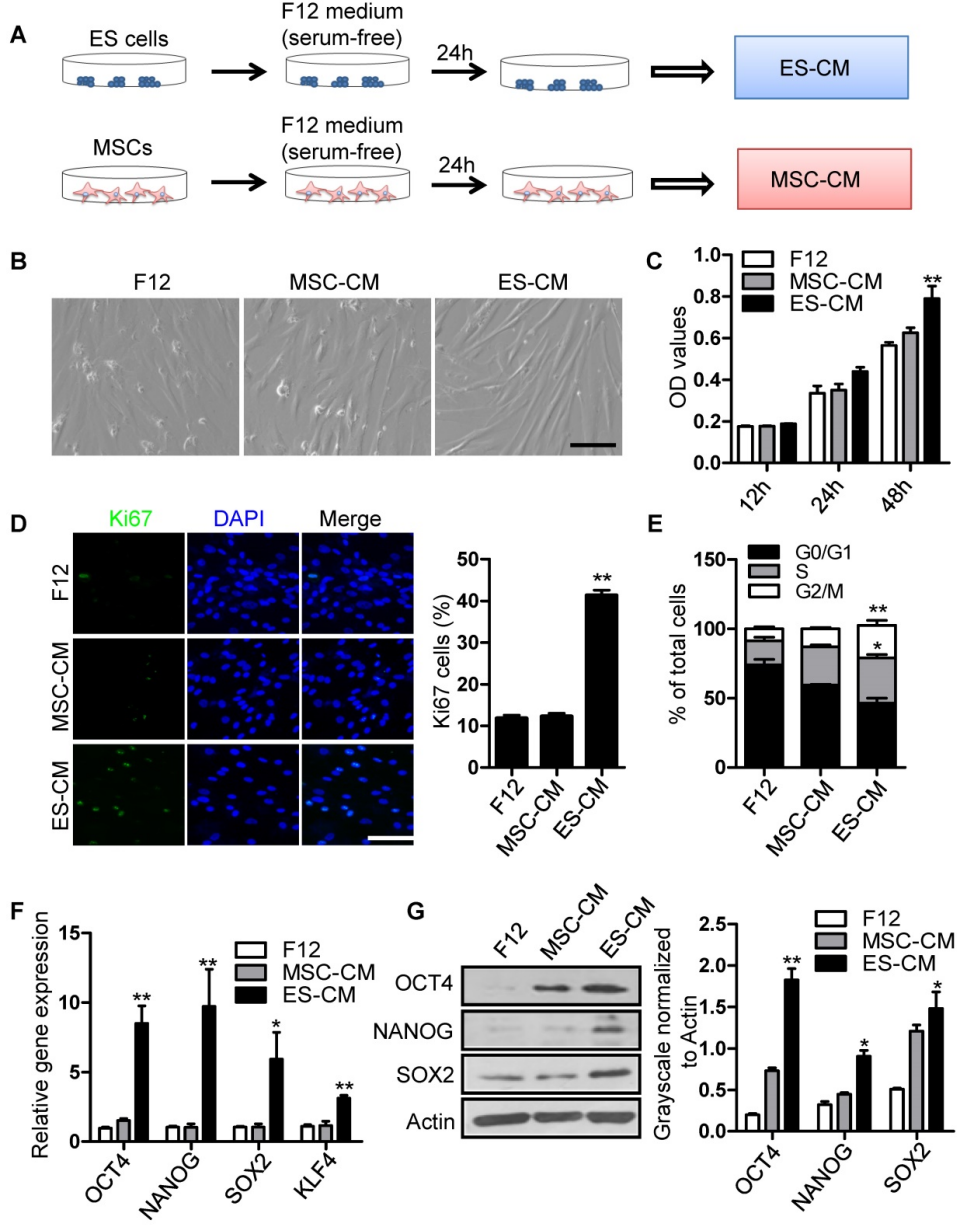
** Effects of ES-CM on the proliferation ability of senescent MSCs. (A)** Schematic representation of the experimental strategy for preparation of conditioned medium. **(B)** Microscopy showed the morphological change of MSCs treated with ES-CM. Scale bar represents 200 μm. **(C)** Effect ES-CM on the proliferation potential of MSCs was analyzed by MTT. **(D)** Immunostaining of Ki67 (left) and the percentage of Ki67-positive cells (right). Scale bar represents 200 um. **(E)** Cell cycle analysis by flow cytometry. **(F)** Real-time PCR analysis the expression levels of stemness-related genes in late-passaged MSCs treated with F12, MSC-CM, and ES-CM for 48h, respectively. **(G)** Western blot analysis the protein levels of OCT4, NANOG and SOX2 in late-passaged MSCs treated with F12, MSC-CM, and ES-CM for 48h, respectively (left). Right panel, quantification of protein levels using ImageJ software, normalized to β-actin. Data are presented as the Mean ± SEM. (n = 3; *p <.05, **p < .01).

To investigate the antisenescence effect of ES cells derived factors on senescent MSCs, we firstly assessed the effect of the ES cells conditioned medium (ES-CM) on senescent MSCs model. ES-CM was obtained from ES cells in DMEM/F12 without FBS and other supplements. Similarly, mesenchymal stem cell conditioned medium (MSC-CM) was obtained from MSCs in DMEM/F12 without FBS and other supplements. The control media was collected under the same conditions without cells **(Figure 1A)**. As mentioned above, P18 MSCs presented aging-related morphologic characteristics **(Figure S1A)**. So P20 MSCs was used as cellular senescence model in further experiments. Using P20 MSCs, we investigated whether ES-CM could rejuvenate the senescence of MSCs. ES-CM treatment rescued the morphology of senescence MSCs to a younger state, which was absent in the control and the MSC-CM group **(Figure 1B)**. In addition to this, ES-CM also promoted the cellular proliferation potential of senescent MSCs analyzed by MTT assay (**Figure 1C)**. The percentage of Ki67 positive cells in the senescent MSCs treated with ES-CM for 48 hours were significantly increased compared to the control medium and MSC-CM treatment, respectively **(Figure 1D)**. Flow cytometry-based cell cycle analysis revealed that ES-CM treatment dramatically increased the S and G2/M phase cell population but reduced the G0/G1 phase **(Figure 1E)**. Collectively, these results demonstrate that ES-CM treatment reactivated proliferation potential of senescent MSCs.

### ES-CM improves the stemness of senescent MSCs

Mesenchymal stem cell, a type of multipotent stem cells from mesoderm, has potential ability of multi-directional differentiation. The stemness of MSCs was impaired by continuously culture *in vitro*. In order to evaluate the effect of ES-CM on stemness of MSC, we further detected the stemness-associated gene expression in late-passaged MSCs treated with different cell conditioned medium. Our data showed that the expression level of SOX2, OCT4, NANOG, KLF4 were increased in MSCs treated with ES-CM for 48 hours **(Figure 1F)**. SOX2, OCT4, NANOG protein levels were also confirmed by western blot, which was significantly increased in ES-CM treated MSCs compared with control and MSC-CM treated MSCs **(Figure 1G)**. Upregulation expression of pluripotent-related genes indicated that ES-CM might enhance stemness of late-passaged MSCs to improve cell self-renewal and pluripotency.

### Antisenescence effect of ES-CM on late-passaged MSCs

**Figure 2:**
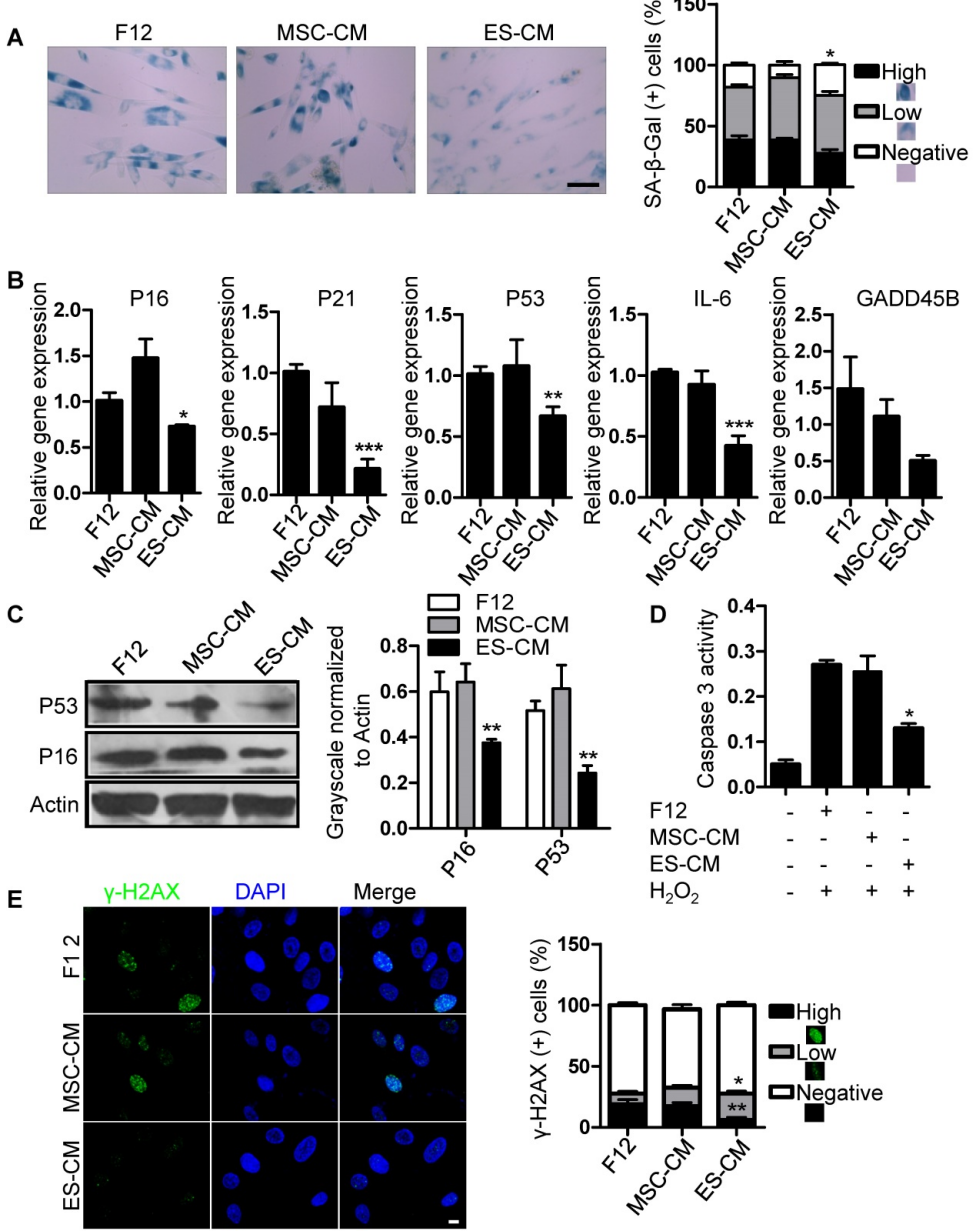
** Antisenescence effects of ES-CM on late-passaged MSCs.** (A) Effects of CM on SA-β-gal activity of late-passaged MSCs (left) and the percentages of SA-β-gal positive cells (right). Scale bar represents 100 um. (B) RT-PCR analysis of stress response genes in late-passaged MSCs treated with F12, MSC-CM, and ES-CM for 48h, respectively. (C) Western blot analysis of protein levels of P16 and P53 in late-passaged MSCs treated with F12, MSC-CM, and ES-CM for 48 h. (D) The activities of caspase 3 in late-passaged MSCs treated with F12, MSC-CM, and ES-CM for 48h (left). Right panel, quantification of protein levels using ImageJ software, normalized to β-actin. (E) DNA damage was analyzed by immunofluorescence staining of γ-H2AX. Scale bar represents 10 μm (left). The percentage of γ-H2AX positive cells was also counted (right). Data are presented as the Mean ± SEM. (n = 3; *p <.05, **p < .01, ***p < .001).

We have shown that the late-passaged MSCs exhibited many features of cellular senescence, such as cell cycle arrest, decreased anti-apoptotic potential, increased DNA damage foci and SA-β-gal activity (**Figure S1, Figure S2**). To determine the antisenescence effects of ES-CM, we treated late-passaged MSCs with control medium, MSC-CM and ES-CM, and examined different cell characteristics associated with cellular senescence. Compared with MSC-CM and control medium, the activity of SA-β-gal in late-passage MSCs treated with ES-CM was significantly decreased, which could be seen as an indicator of cellular senescence **(Figure 2A)**. We also evaluated the expression of senescence-associated genes, including P16, P21, P53, GADD45B, and IL-6, which were all down-regulated in late-passaged MSCs treated with ES-CM **(Figure 2B)**. ES-CM treatment also decreased the protein levels of P16 and P53 analyzed by western blot **(Figure 2C)**. Senescent cells have their own unique secretory phenotype (SASP), including inflammatory cytokines (IL-1, IL-6, IL-8), growth factors (HGF, TGF) and proteases (MMP1, MMP3, MMP9, and TIMP2) 25. In late-passaged MSCs, ES-CM treatment effectively decreased the production of matrix metalloproteinase 9 (MMP9) to alleviate senescence-associated secretary phenotype (SASP) **(Figure S3A)**.

Senescent MSCs display cell cycle arrest and decreased anti-apoptosis 26, 27. We hypothesized that ES-CM contribute to the apoptotic resistance of senescent MSCs. Next, we detected the effects of ES-CM on cell cycle and apoptosis of late-passage MSCs. The percentage of apoptotic cells was lower in MSCs treated with ES-CM (4.34%) than in the MSCs treated with control medium (8.56%) and MSC-CM (7.18%) **(Figure S3B).** We also examined the activity of caspase 3 (an apoptotic executioner caspase) and found that ES-CM treatment significantly reduced caspase 3 activity **(Figure 2D)**, which indicating that the apoptosis in late-passaged MSCs was alleviated by ES-CM treatment. In the control group, the positive ratio of γ-H2AX is about 19% and decreased to 6% after the treatment of ES-CM for 48 hours. However, no significant changes were observed in the MSC-CM group. The number of γ-H2AX foci was also decreased in the group of ES-CM treatment **(Figure 2E)**. The decrease in the number of γ-H2AX foci indicated that ES cells derived factors could attenuate the DNA damage response (DDR) of senescent MSCs. Taken together, our data strongly suggest that factors secreted by ES cells may have antisenescence effects on the late-passaged MSCs.

### The antisenescence effects of ES-EVs on late-passaged MSCs

**Figure 3:**
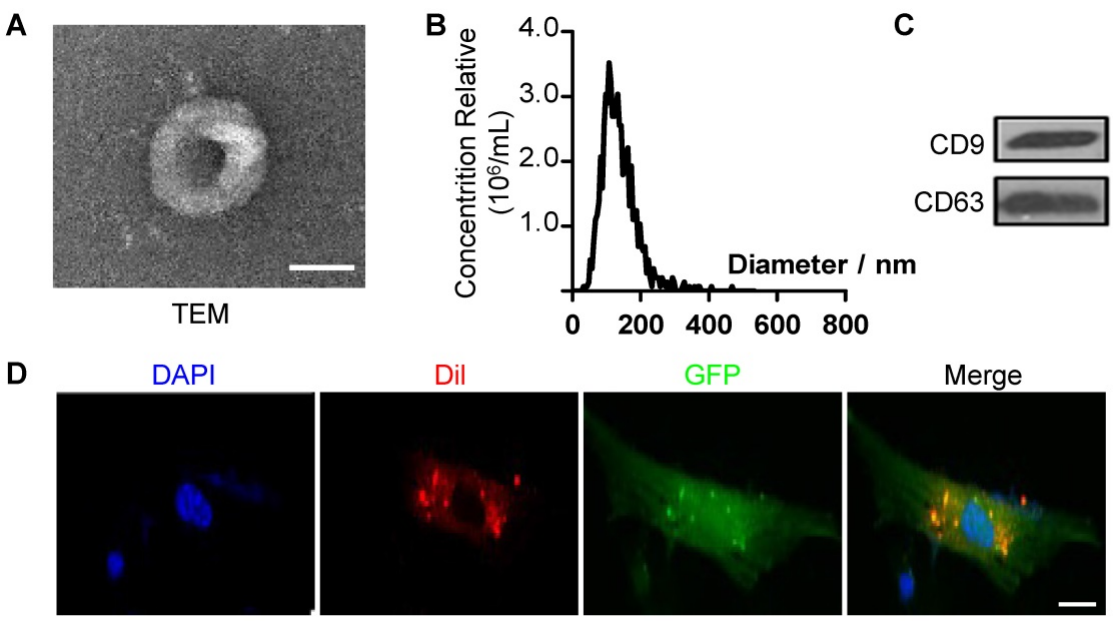
** Characteristics of extracellular vesicles derived from ES cells.** (A) TEM image of ES-EVs. Scale bar, 100 nm. (B) Size distribution of ES-EVs was measured by dynamic light scattering. (C) The expression of CD9 and CD63 in ES-EVs was analyzed using western blotting. (D) Internalization of ES-EVs was analyzed by immunofluorescence detection. Dil-labeled exosomes (red) was detected in the MSCs which expressing green fluorescent protein (GFP, green). Scale bar, 10 μm.

To further explore the mechanism of antisenescence effect of ES-CM, we isolated extracellular vesicles (EVs) from the ES-CM (named ES-EVs). We firstly characterized the ES-EVs using transmission electron microscope (TEM), dynamic light scattering analysis and western blotting. The TEM image showed that the particle pellets were round-shaped vesicles with the membrane bounded **(Figure 3A)**. As shown in the dynamic light scattering analysis, the average diameter of ES-EVs was 100 nm **(Figure 3B)**. EVs membranes were enriched with endosome-specific tetraspanins (such as CD9, CD63, and CD81) according to the latest guideline published on Journal of Extracellular Vesicles 28. CD9 and CD63 were detected in ES-EVs using western blotting analysis (**Figure 3C**). To detect whether the ES-EVs were internalized by MSCs, ES-EVs were labeled with Dil dye (red) and incubated with GFP expressing MSCs *in vitro*. After 6 hours of incubation, the Dil co-located with GFP, indicating that the Dil labeled ES-EVs were taken up by MSCs **(Figure 3D)**. All of results above demonstrated that ES-EVs were successfully internalized by MSCs.

**Figure 4:**
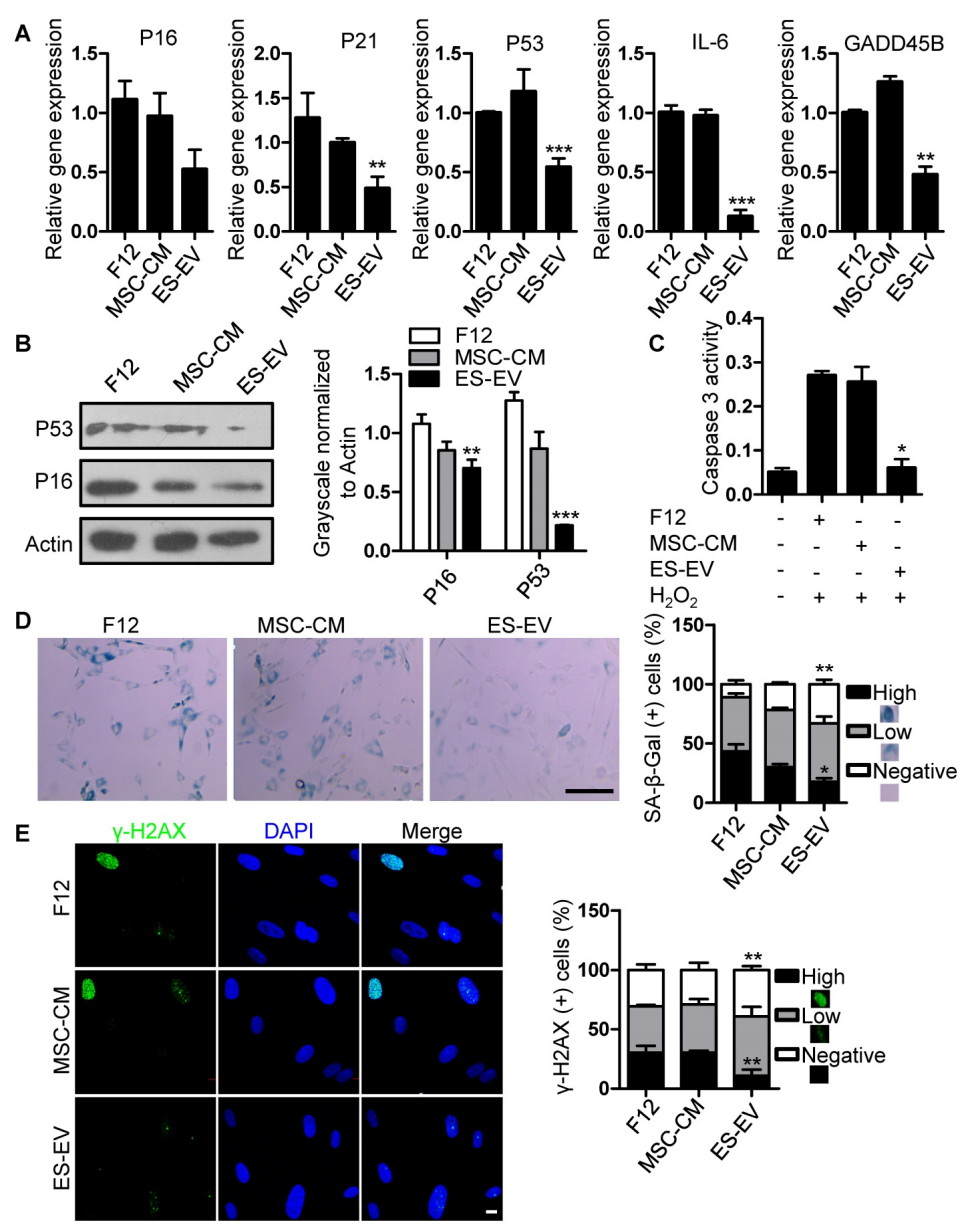
** Antisenescence activity of ES-EVs.** (A) RT-PCR analysis of the expression levels of senescence-associated genes in late-passaged MSCs treated with F12, MSC-CM, and ES-EVs for 48h respectively. (B) Protein levels of P16 and P53 were detected by western blotting. Right panel, quantification of protein levels normalized to β-actin. (C) The activities of caspase 3 in late-passaged MSCs treated with F12, MSC-CM, and ES-EVs for 48h respectively. (D) Effects of ES-EVs on SA-β-gal activity of MSCs and the percentage of SA-β-gal-positive cells. Scale bar represents 200 μm. (E) DNA damage foci γ-H2AX was detected by immunofluorescence staining. Scale bar represents 10 μm. Data are presented as the Mean ± SEM. (n = 3; *p <.05, **p < .01, ***p < .001).

Next, we detected the antisenescence effects of ES-EVs on late-passaged MSCs. Firstly, we detected the proliferation potential of senescent MSCs with series of concentration of ES-EVs, and found 100 μg/mL has highest proliferation potential to the senescent MSCs. So, the concentration of 100 μg/mL was used in the following experiments. Then we evaluated the expression of senescence-associated genes as mentioned above, and found that the expression levels of P16, P21, P53, GADD45B, and IL-6, were all decreased in MSCs treated with ES-EVs compared with the control group **(Figure 4A)**. In addition, ES-EVs treatment significantly decreased the protein levels of both P53 and P16 in senescent MSCs** (Figure 4B).** The activity of caspase 3 was also decreased in senescent MSCs treated with ES-EVs **(Figure 4C)**. Treatment of late-passaged MSCs with ES-EVs (100 μg/mL) for 48 hours also reduced SA-β-gal activity (the percentage of high positive is 18%) compared to the control group (43%) **(Figure 4D)**. Our previously work showed ES-CM might alleviate DNA damage response in senescent MSCs. In senescent MSCs treated with ES-EVs the percentage of γ-H2AX-positive cells were also decreased compared to control group **(Figure 4E).** These results showed that ES-EVs also significantly rejuvenated the late-passaged MSCs.

To further explore whether ES-EVs have antisenescence effects on early-passaged of MSCs (P10 MSC), using MTT assay, we examined the proliferation ability of P10 MSCs, which was pretreated with F12, MSC-CM, ES-EVs, respectively. The results showed that ES-EVs also improved the proliferation ability of early-passaged MSCs at 48 h **(Figure S4A).** We further analyzed the expression levels of stemness-associated genes in early-passage MSCs treated with ES-EVs. Our data showed that the expression levels of SOX2, OCT4, NANOG, and KLF4 were increased in early-passaged MSCs treated with ES-EV for 48 hours **(Figure S4B)**, accompanying by a lower level of SA-β-gal activity **(Figure S4C, S4E)**. We evaluated the expression levels of senescence-associated genes and found that the expression levels of P16, P21, P53, GADD45B, and IL-6, were also decreased in MSCs treated with ES-EVs (**Figure S4D**). These results demonstrated that the ES-EVs also have antisenescence effects even on the young MSCs (early-passaged MSCs).

### ES-EVs enhanced the therapeutic effect of senescent MSCs *in vivo*

**Figure 5:**
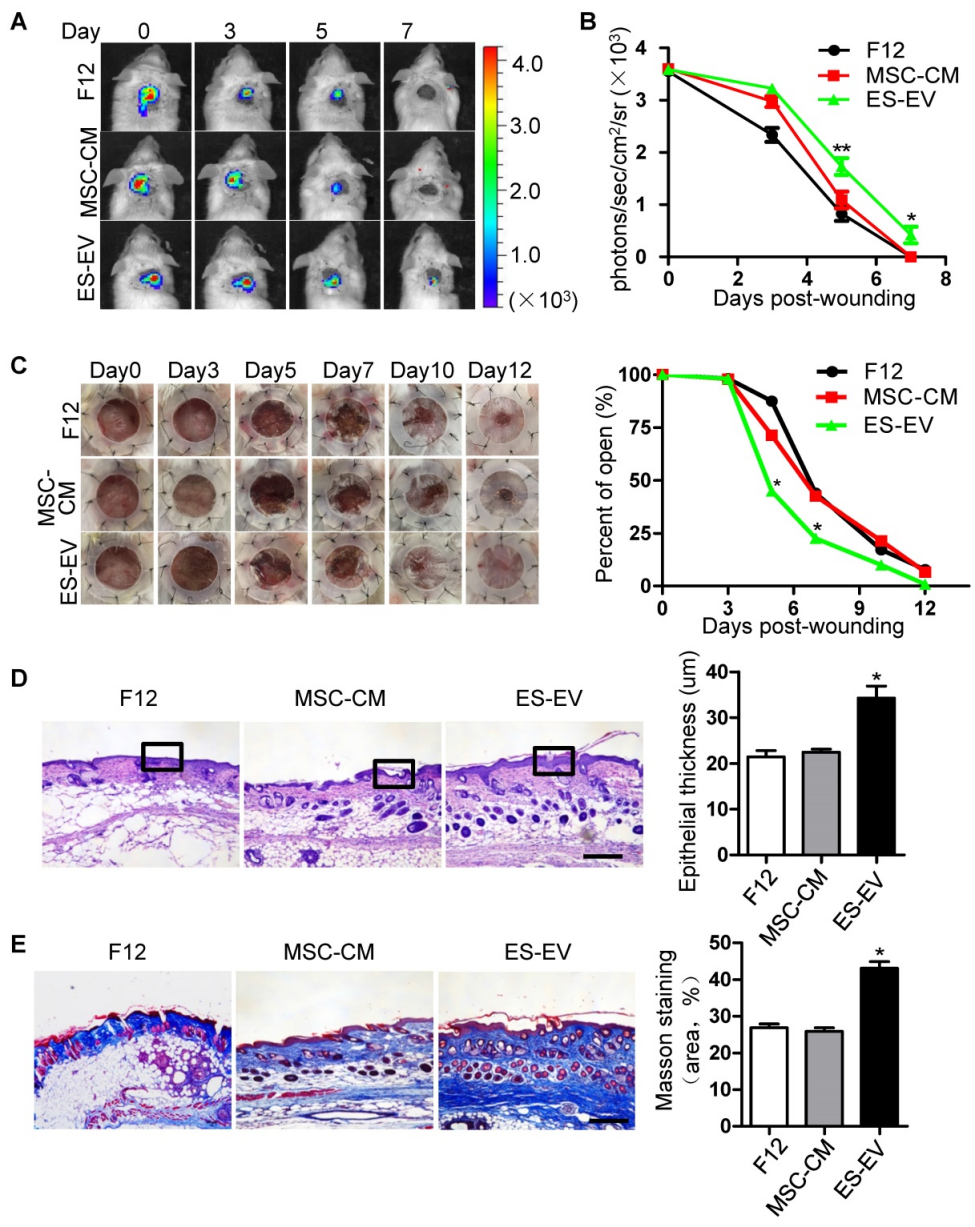
** Enhanced wound-healing process of senescent MSCs by ES-EVs.** (A) The fate of MSCs after transplantation was tracked by molecular imaging. Images were from representative animals receiving 5×10^5^ MSCs, which was pretreated with F12, MSC-CM or ES-EVs respectively. (B) Quantitative analysis of BLI signals demonstrate that cell survival was improved by ES-EVs at all time points. (C) Analysis of the wound-healing area at different time poins (left) and the quantitative analysis of wound-healing area (right). (D) Histologic analysis of wound area by HE staining. Scale bar represents 100 μm. (E) Histologic analysis of wound area by Massion trichrome, Scale bar represents 100 μm. Data are presented as the Mean ± SEM. (n = 3; *p <.05, **p < .01).

MSCs have an signaficant effect on wound-healing and some other diseases 29, 30. To investigate whether ES-EVs could enhance the therapeutic effects of MSCs *in vivo*, luciferase-labeled senescent MSCs were pre-treated with ES-EVs, and implanted into the injury area of cutaneous wound mice model. The cells were next determined by measuring luminescence signals within 7 days. In line with the observed repression of cellular decay* in vitro*, ES-EVs treatment also effectively restored the viability of senescent MSCs *in vivo*** (Figure 5A)**. On day 5 and 7, in the ES-EVs treatment group, the amount of viable cells at the injury site was significantly higher than in that of control groups **(Figure 5B)**. We also tested the effect of senescent MSCs with different treatment on wound healing *in vivo*, and found that ES-EVs treatment improved the healing process of a full-thickness excisional skin wound-healing model compared with control group **(Figure 5C)**. On day 12 the wound had been healed in ES-EVs treatment, but which were not in other two groups **(Figure 5C)**. Histologic analysis of the wounded areas demonstrated that ES-EVs treatment enhanced the thickness of epithelium as well as promoted the synthesis and regeneration dermal collagen **(Figure 5D, 5E)**. Even for the early-passaged MSCs (young MSCs), pretreated with ES-EVs aslo improved its therapeutic effects *in vivo* **(Figure S5)**. Taken together, these data indicated that ES-EVs enhanced the therapeutic effects of MSCs *in vivo*, by increasing epithelial and dermal cell proliferation, angiogenesis, dermal collagen synthesis, and further accelerate skin wound healing.

### Involvement of IGF1/PI3K/AKT pathway in the antisenescence activities of ES-EVs

**Figure 6:**
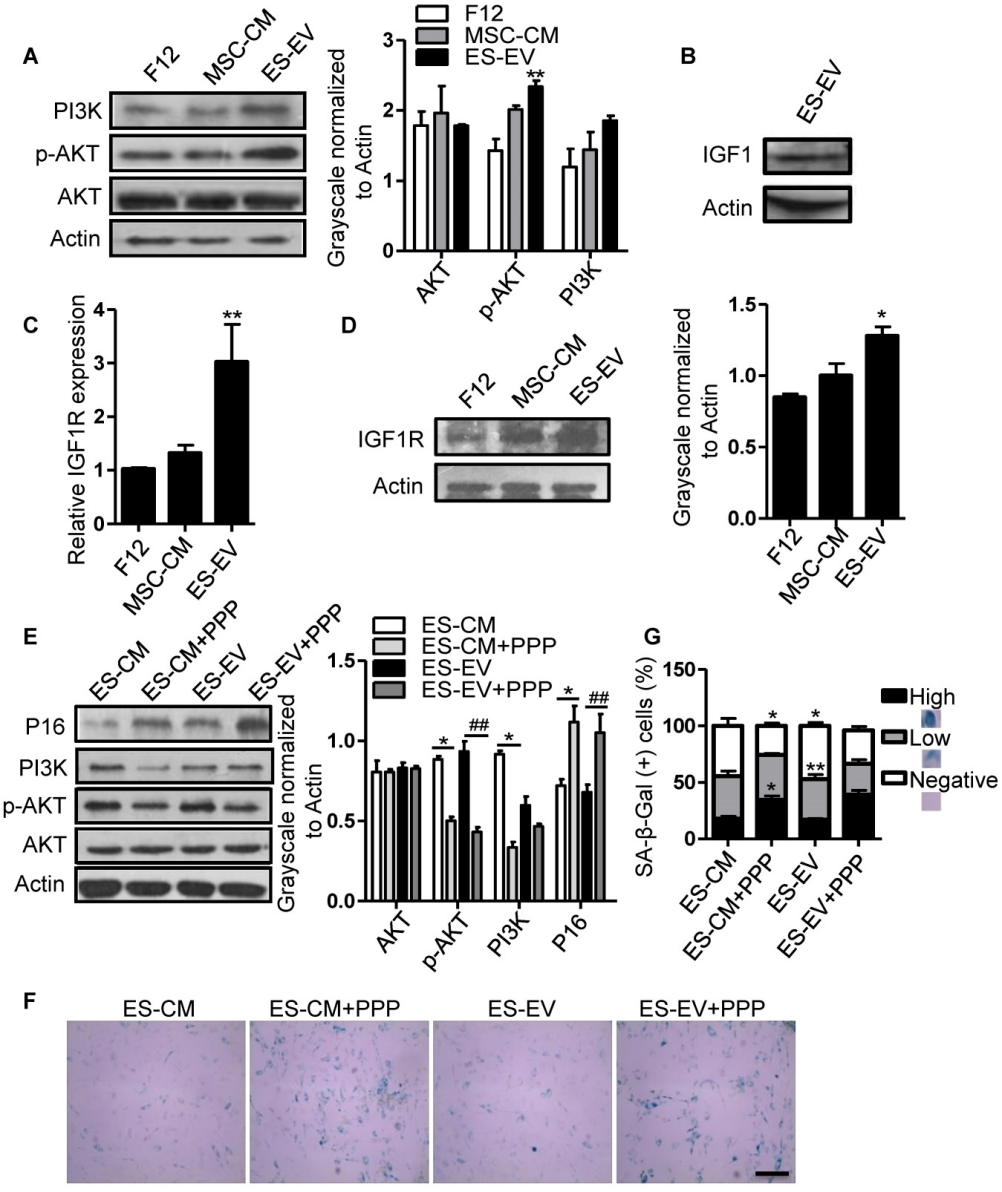
** ES-EVs activate the IGF1/PI3K/AKT pathway in senescent MSCs.** (A) Expression levels of PI3K, AKT, and p-AKT in MSCs with different treatment were detected by western blot (left). Histogram showed the quantitative analysis of western blot (right). (B) IGF1 was detected in ES-EVs using western blot analysis. (C) RT-PCR analysis the expression of IGF1R in late-passaged MSCs treated with F12, MSC-CM, and ES-EVs for 48 hours. (D) Expression level of IGF1R in MSCs with ES-EVs treatment for 48 hours was detected by western blot. (E) Protein levels of P16, PI3K, AKT, and p-AKT in late-passaged MSCs treated with picropodophyllin (PPP: IGF1R inhibitor) were analyzed using western blot (left). Histogram showed the quantitative analysis of western blot (right). (F) Effect of PPP on SA-β-gal activity of late-passaged MSCs. Scale bar represents 100 μm. (G) Histogram showed the quantitative analysis of the percentage of SA-β-gal-positive cells. Data are presented as the Mean ± SEM. (n = 3; *p <.05, **p < .01).

Insulin-like growth factor (IGF) plays a crucial role in cellular senescence and many other cellular processes, including growth, proliferation, survival, development and canceration 31. IGF1 activates PI3K/AKT pathway by binding to its receptor IGF1R, thereby increasing cell survival and promoting growth and proliferation 32. Several studies have shown that IGF1/PI3K/AKT signaling also plays a key role in promoting cell proliferation and cellular senescence 33-36. So, we further analyzed the role of this pathway in the antisenescence effect of ES-EVs on late-passaged MSCs. We firstly detected the activation of PI3K/AKT pathway in late-passaged MSCs with different treatment and found that the ES-EVs could activate PI3K/AKT pathway. The level of p-AKT was higher in ES-EVs treatment group than that in other two groups **(Figure 6A)**. IGF1 was also detected in ES-EVs. These results strongly indicated the IGF1-PI3K pathway might be involved in the antisenescence effect of ES-EVs **(Figure 6B)**. To verify this hypothesis, we also analyzed the expression level of IGF1R and found that IGF1R increased in senescent MSCs after treated with ES-EVs for 48 hours by real-time PCR and western blotting analysis **(Figure 6C, 6D)**, which further suggested ES-EVs activated IGF1/PI3K/AKT pathway. To confirm the result above, we employed the picropodophyllin (PPP), an inhibtor of IGFR, to suppress IGF1/PI3K/AKT pathway. The result indicated that IGF1/PI3K/AKT pathway was significantly suppressed with the treatment of PPP **(Figure 6E)**. As a result, almost all of the senescence-associated characteristics were almost be rescured in the PPP group, such as up-regulation of P16 expression **(Figure 6E)** and increased activity of SA-β-gal **(Figure 6F, 6G)**, compared with the group without PPP.

**Figure 7:**
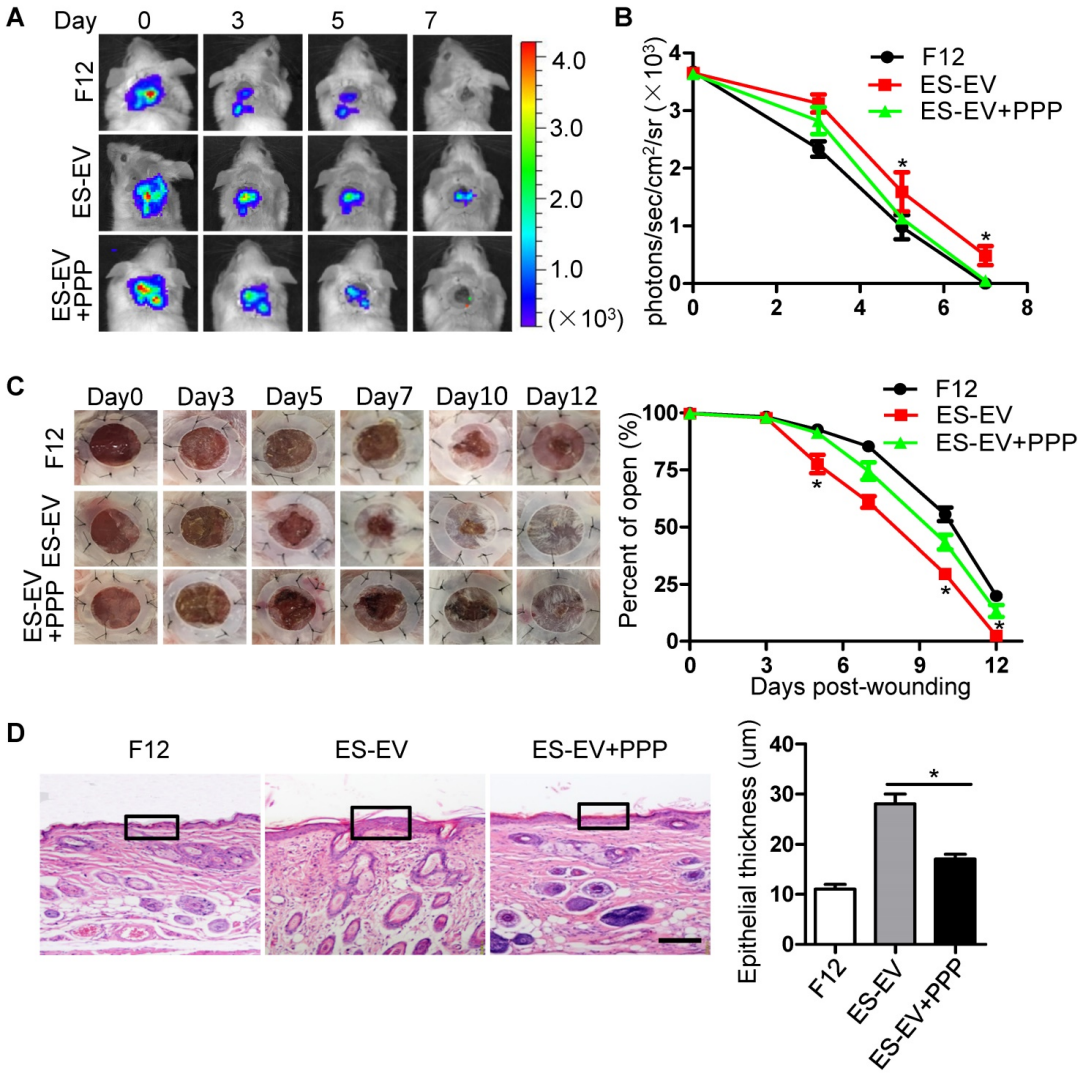
** Inhibition of IGF1/PI3K/AKT pathway attenuates the *in vivo* effects of ES-EVs on senescent MSCs.** (A) The fate of MSCs after transplantation was tracked by molecular imaging. Images were from representative animals receiving 5×10^5^ MSCs with F12, ES-EVs, or ES-EVs and PPP. (B) Quantitative analysis of BLI signals. (C) Analysis of the wound-healing area at different time points (left). Quantitative analysis of wound-healing area (right). (D) Histologic analysis of wound area by HE staining. Scale bar represents 50um. Data are presented as the Mean ± SEM. (n = 3; *p <.05).

The results above suggested that the inactivation of IGF1/PI3K/AKT pathway offset the antisenescence effects of ES-CM and ES-EVs on aging MSCs *in vitro*. Next, we further investigated whether the inhibition of IGF1-AKT pathway could attenuate the antisenescence effect of ES-EVs on the aged MSCs *in vivo*. We compared the therapeutic effect of senescent MSCs that pretreated with ES-EVs in the present of PPP (IGF1R inhibitor) or not. Luciferase-labeled senescent MSCs implanted into the injured area were determined by measuring luminescent signals within 7 days. The result showed that PPP significantly attenuated the effect of ES-EVs on the senescent MSCs* in vivo* **(Figure 7A, 7B)**. PPP treatment also slowed the healing process of a full-thickness excisional skin wound-healing model compared to the ES-EVs group **(Figure 7C)**. In the group of ES-EVs with PPP, the thickness of epithelium in the wounded area was also thinner than in the ES-EVs group **(Figure 7D)**. These results suggested that the antisenescence effect of ES-EVs was mediated by the IGF1 signal activation**.**

## Discussion

**Figure 8:**
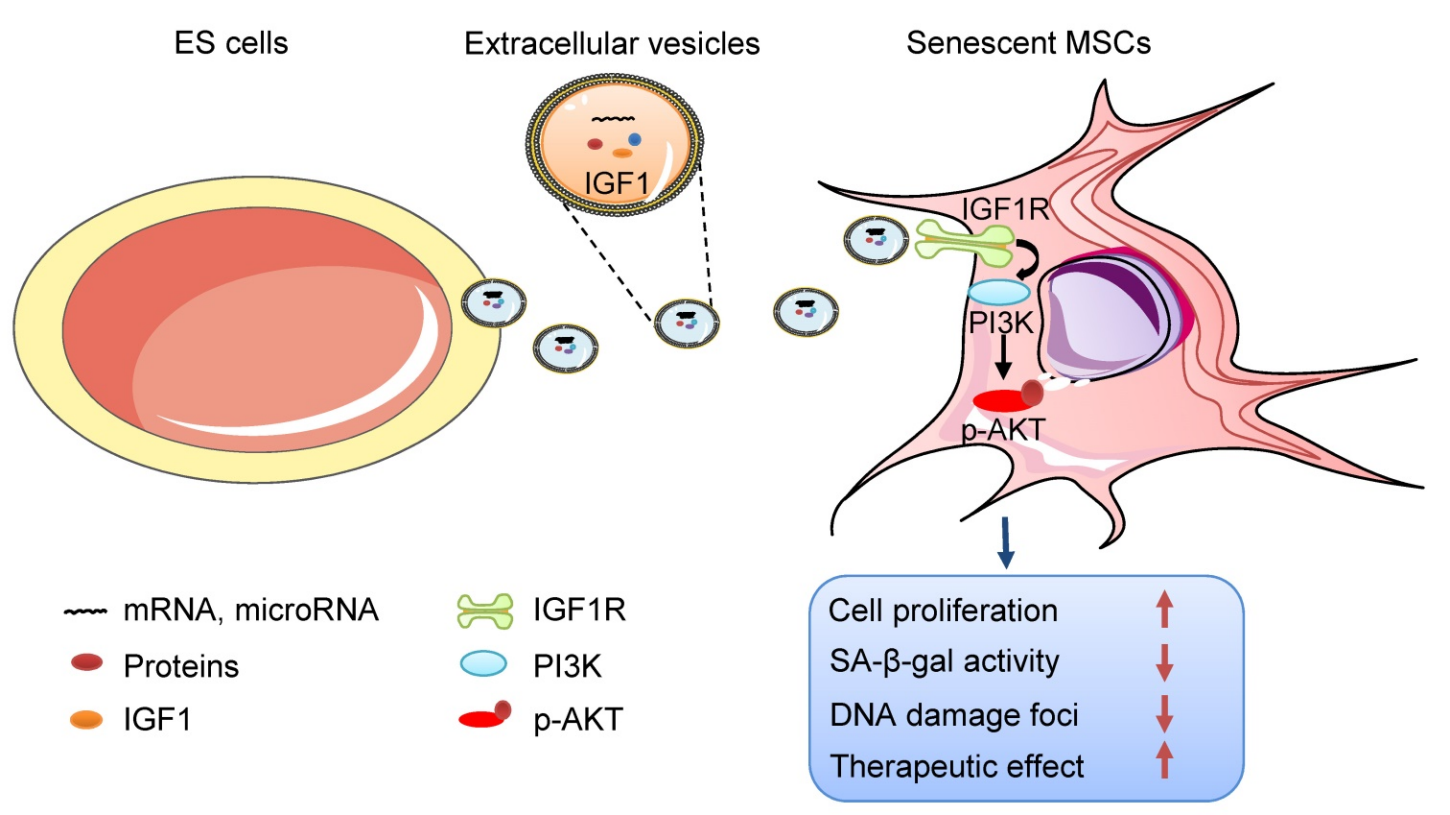
** Schematic illustration the role of ES-EVs on MSCs.** The ES-EVs transfer the IGF1, a secreted factor derived from ES cells, to senescent MSCs and activate the IGF1R/AKT signaling pathway of MSCs. Then mediating ES-EVs enhances the therapeutic effect of MSCs by improving cellular proliferation, increasing stemness, suppressing the senescence phenotypes, decreasing SA-β-gal activity, and reducing DNA damage.

In this study, we focused on the effects of ES-EVs on the senescent MSCs. Our data demonstrated that ES-EVs have antisenescence activity on MSCs. Specifically, ES-EVs enhanced the proliferative potential, decrease the SA-β-gal activity, enhance the stemness, decreased the DNA damage foci, and decreased the expression levels of P16 and P53. We further investigated the factors that mediate the antisenescence activity of ES-CM and found that the extracellular vesicles exerted antisenescence effects through upregulating the expression of IGF1R subsequently activating the PI3K/AKT pathway in senescent MSCs. In addition, ES-EVs markedly enhanced the retention of MSCs in the mouse cutaneous wound sites and facilitated the cutaneous wound healing process **(Figure 8).**

Many studies have shown that MSCs offer great promise for regenerative therapy and tissue engineering, because they have significantly less immune responses 37, 38, less ethical controversies and less tumorigenic risks. Thus, MSCs provide great promise for regenerative therapy, tissue engineering, beauty and anti-aging. MSCs need to be maintained in youthful state with the optimized culture conditions that support their self-renewal and multipotent properties. Although the senescence is unavoidable, it has been found that the cellular senescence rate and process could be delayed by secretory factors and small molecules 39. Circulating factors derived from young cells can restore a youthful state of senescence cells 40. Rapamycin, a well-known mTOR inhibitor 41, is the most common drug used to treat patients with Hansen disease 42. Urolithin A also has been found have anti-aging effects on replicative senescent human skin fibroblasts 43.

Human ES cells and mouse ES cells are derived from blastocyst-stage embryos, and posses the remarkable property of pluripotency and give rise to all cells of the origanism 44. For this purpose, ES cells are thought to hold great promise for regenerative medicine 44. Two different sources of ES cells have some biological and epigenetic characteristics in common, such like growth properties, X-chromosome activation state, the gene expression profile and the related signaling pathways 45, 46. Research also found that the genomic distribution is very similar in both mouse ES cells and human ES cells, such as some novel transcriptional regulators and epigenetic signatures 47. Therefore, the same components maybe exist in the extracellular vesicles derived from human and mouse ES cells. In our study, the MSCs treated with ES-EVs were used to treat mouse cutaneous wound, not the ES-EVs. This treatment strategy circumvents the therapeutic risk of ES cells in the application. On top of all these, extensive differences still exist between human and mouse ES cells. Human ES cells are considered to be more closely to resemble mouse epiblast stem cells (mEpiSCs) that are derived from the post-implantation epiblast 48, 49.

Although ES cells hold a great promise for the regenerative medicine, their ethical and tumorigenic potential limite the clinical application. One study found that the the conditioned medium from mouse ES cells have an effectively antisenescence effect on senescent human dermal fibroblasts 13. The self-renewal ability and some functions of stem cells are known to decline with advancing aging. The senescent MSCs might participate in the acceleration of pathologies such as obesity, degenerative diseases, and cancers. Recent studies found EVs secreted by human induced pluripotent stem cell (iPSCs) could alleviate aging cellular phenotypes of senescent MSCs and aged human dermal fibroblasts 50, 51. However, the antisenescence effect of ES-EVs on senescent MSCs have not been elucidated. Herein, we firstly identify that ES-EVs can siginificantly rejuvenante the senescent MSCs, and effectively ehanced the therapeutic effect of MSCs *in vivo*.

Regarding of the molecular mechanism, we found that treatment with ES-EVs effectively activated the IGF/PI3K/AKT pathway in senescent MSCs. IGF1 receptor is a cell surface receptor tyrosine kinase that can bind its cognate ligands IGF1 and IGF2 to activate the principle downstream PI3K/AKT signaling pathways, which promote cell proliferation, differentiation, migration, survival and inhibit apoptosis 36, 52, 53. The proliferation and multipotent of MSCs can be increased through activating IGF1R signaling or low-oxygen tension 35. Many studies found that decreasing IGF1 were likely related to the life-prolonging effects in aged individual. However, there also have some different points of view that IGF1 rescued cellular senescence 54-57. Given that the complex components in ES-EVs, other factors might have effects in rejuvenating cells as well, such as TGF 58-60. We will further investigate the components of ES-EVs and the other mechanisms of ES-EVs in rejuvenating in the future research. Taken together, our finding showed that the ES-EVs not only can maintain the self-renewal and multipotent properties of MSCs but also as an effective substance for enhancing the therapeutic effects of MSCs.

## Conclusion

In summary, our data suggest that ES-EVs can effectively rescue the senescence-associated phenotypes of MSCs by enhancing proliferation potential, increasing the stemness, suppressing the expression of senescent-related genes, decreasing SA-β-gal activity and DNA damage. ES-EVs further improve the therapeutic effect of MSCs *in vivo* and accelerate the mouse skin wound-healing process. The antisenescence effect of ES-EVs on MSCs is mediated by IGF1/PI3K/AKT signaling pathway. ES-EVs as a pretreatment factor can be used as an excellent substance to enhance the therapeutic effect of MSCs.

## Supplementary Material

Supplementary figures and tables.
