# Supplementary material for: Embryonic stem cell-derived extracellular vesicles enhance the therapeutic effect of mesenchymal stem cells
Source: Theranostics. 2019 Sep 21;9(23):6976–90. doi: 10.7150/thno.35305 (PMC6815953; doi:10.7150/thno.35305)
Supplement: Supplementary file 1 — Supplementary figures and tables. [file thnov09p6976s1.pdf]

Supporting Information:

Embryonic stem cell-derived extracellular vesicles enhance the therapeutic effect of mesenchymal stem cells

Yan Zhang[1, 2], Jia Xu[1, 2], Siying Liu[1, 2], Meikuang Lim[5], Shuang Zhao[2, 3], Kaige Cui[1, 2], Kaiyue Zhang[1, 2], Lingling Wang [3], Qian Ji[4], Zhongchao Han[5], Deling Kong[2], Zongjin Li[1, 2]✉, and Na Liu[1, 2]✉

1. School of Medicine, Nankai University, Tianjin, 300071, China

2. Key Laboratory of Bioactive Materials, Ministry of Education, College of Life Sciences Nankai University, Tianjin, 300071, China

3. College of Life Sciences, Nankai University, Tianjin, 300071, China

4. Department of Radiology, Tianjin First Central Hospital, Tianjin, China; 24 Fukang Road, Nankai District, Tianjin, 300071, China

5. Beijing Engineering Laboratory of Perinatal Stem Cells, Beijing Institute of Health and Stem Cells, Health & Biotech Co., Beijing, 100176, China

✉Corresponding authors: Na Liu, Email: liuna@nankai.edu.cn; Zongjin Li, Email: zongjinli@nankai.edu.cn

Table S-1. Primers for real-time PCR assay

Gene Name	Primers
OCT4	Forward: GCTCGAGAAGGATGTGGTCC<br>Reverse: CGTTGTGCATAGTCGCTGCT
SOX2	Forward: CACTGCCCCTCTCACACATG<br>Reverse: TCCCATTTCCCTCGTTTTTCT
NANOG	Forward: ACAACTGGCCGAAGAATAGCA<br>Reverse: GGTTCCCAGTCGGGTTCAC
KLF4	Forward: AGCCACCCACACTTGTGACTAT<br>Reverse: AGTGGTAAGGTTTCTCGCCTGT
P16	Forward: GAGCAGCATGGAGCCTTC<br>Reverse: CCTCCGACCGTAACTATTCG
P21	Forward: TGTCCGTCAGAACCCATGC<br>Reverse: AAAGTCGAAGTTCCATCGCTC
P53	Forward: CAGCACATGACGGAGGTTGT<br>Reverse: TCATCCAAATACTCCACACGC
IL6	Forward: CCTTCACTCCATTCGCTGTCT<br>Reverse: TGTCGACCATGCGCTTAATG
GADD45B	Forward: AGACAGGACCCCCGAAGCT<br>Reverse: GGGCTTGCAGTCAGTCTCACT
IGF1R	Forward: AATAAGCCCCCAAAGGAATG<br>Reverse: TGGCAGCACTCATTGTTCTC
ACTIN	Forward: CATGTACGTTGCTATCCAGGC<br>Reverse: CTCCTTAATGTCACGCACGAT

Supporting Figures

Figure S-1

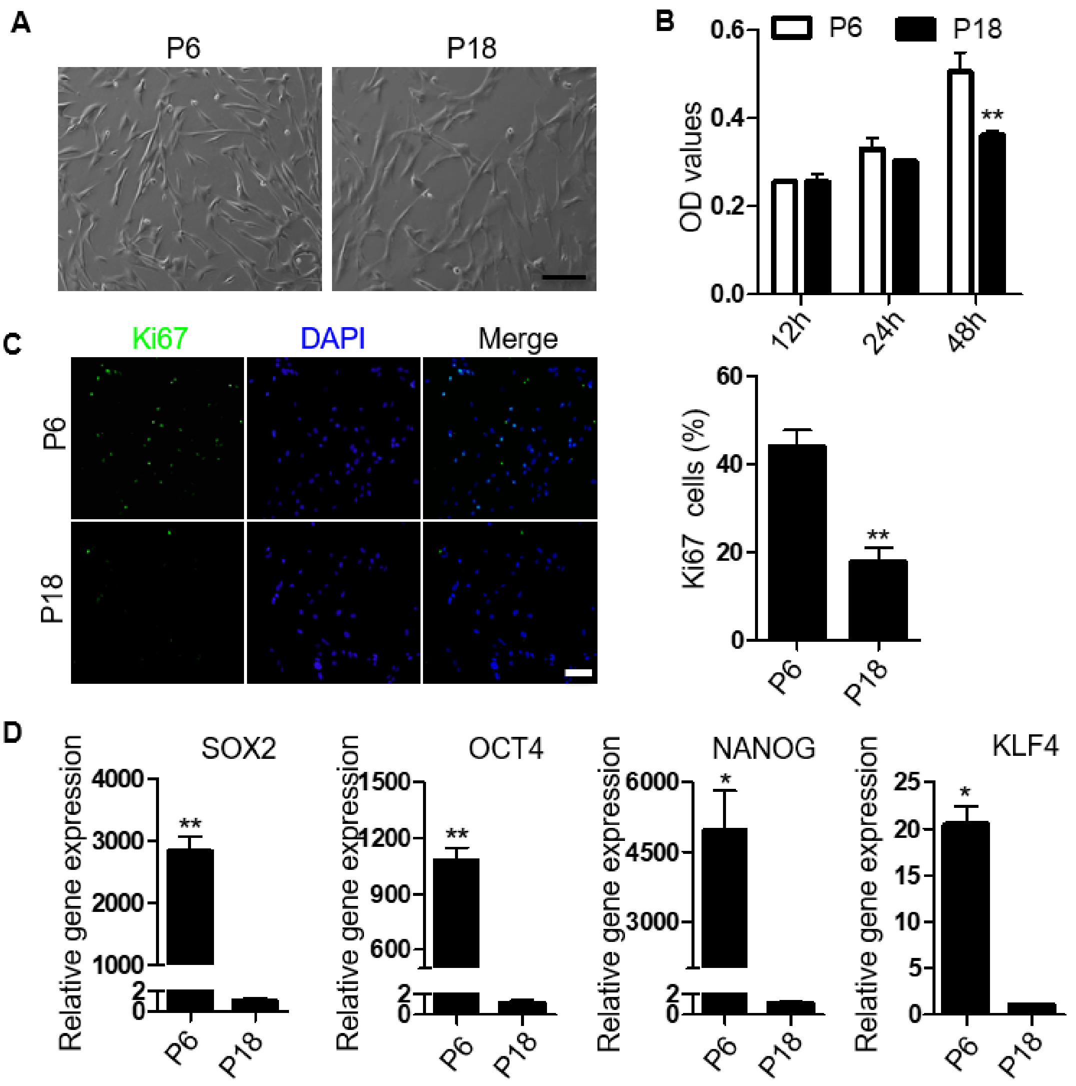

Figure S-1. The proliferation and stemness were decreased in late-passaged MSCs. (A) Microscopy showed a morphological change in P6 and P18 MSCs. Scale bar represents 200 μm. (**B)** The proliferation of P6 and P18 MSCs was measured by MTT. (**C)** Representative image showed the proliferation marker of Ki67 (green) in the P6 and P18 MSCs and quantification of the percentage of Ki67 positive MSCs. Scale bar, 100 μm. (**D)** RT-PCR analysis of stemness-related gene expression in P6 and P18 MSCs. Data are presented as the Mean ± SEM. (n = 3; *p <.05, **p < .01).

Figure S-2

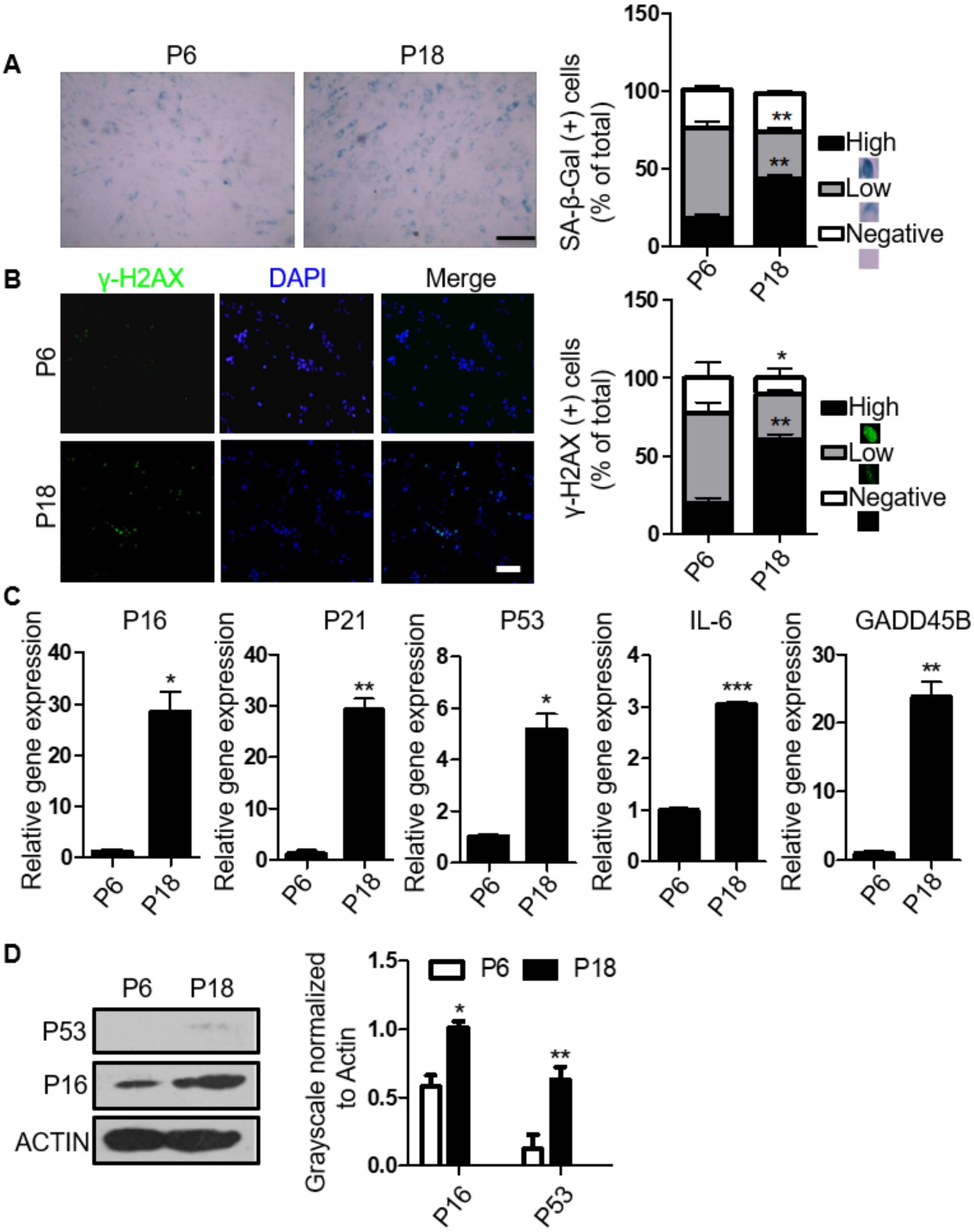

Figure S-2. Cell senescence of late-passaged MSCs. (**A)** SA-β-gal activity staining of P6 and P18 MSCs and the percentages of SA-β-gal positive cells. (**B)** Immunofluorescence staining of γ-H2AX. Scale bar, 100 μm**. (C)** Analyzed of the expression of stress response genes in the p53 pathway, senescence-associated metalloprotease, and interleukin-6 using qPCR. (**D)** Expression level of P16, P53 by western blot. Data are presented as the Mean ± SEM. (n = 3; *p <.05, **p < .01, ***p <.001).

Figure S-3

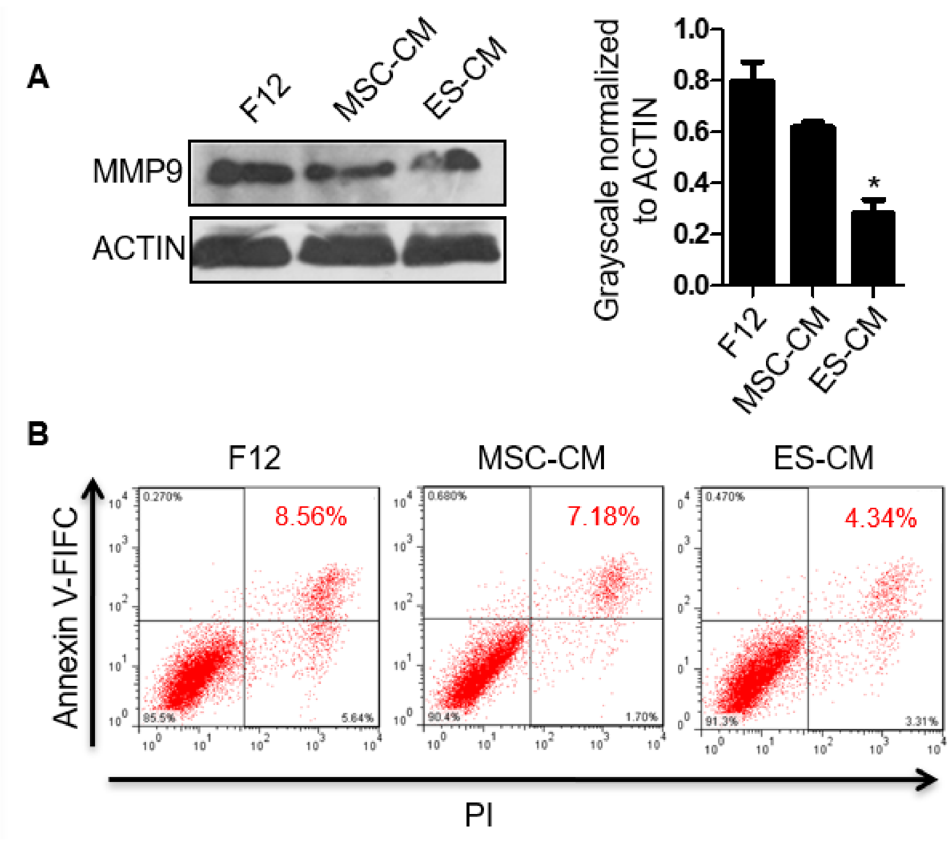

Figure S-3. Antisenescence effects of ES-CM on late-passaged MSCs. (A) Analyzed of senescence-related secretory phenotype (MMP9) by western blot. **(B)** Analysis of cell apoptosis by flow cytometry. Data are presented as the Mean ± SEM. (n = 3; *p <.05).

Figure S-4

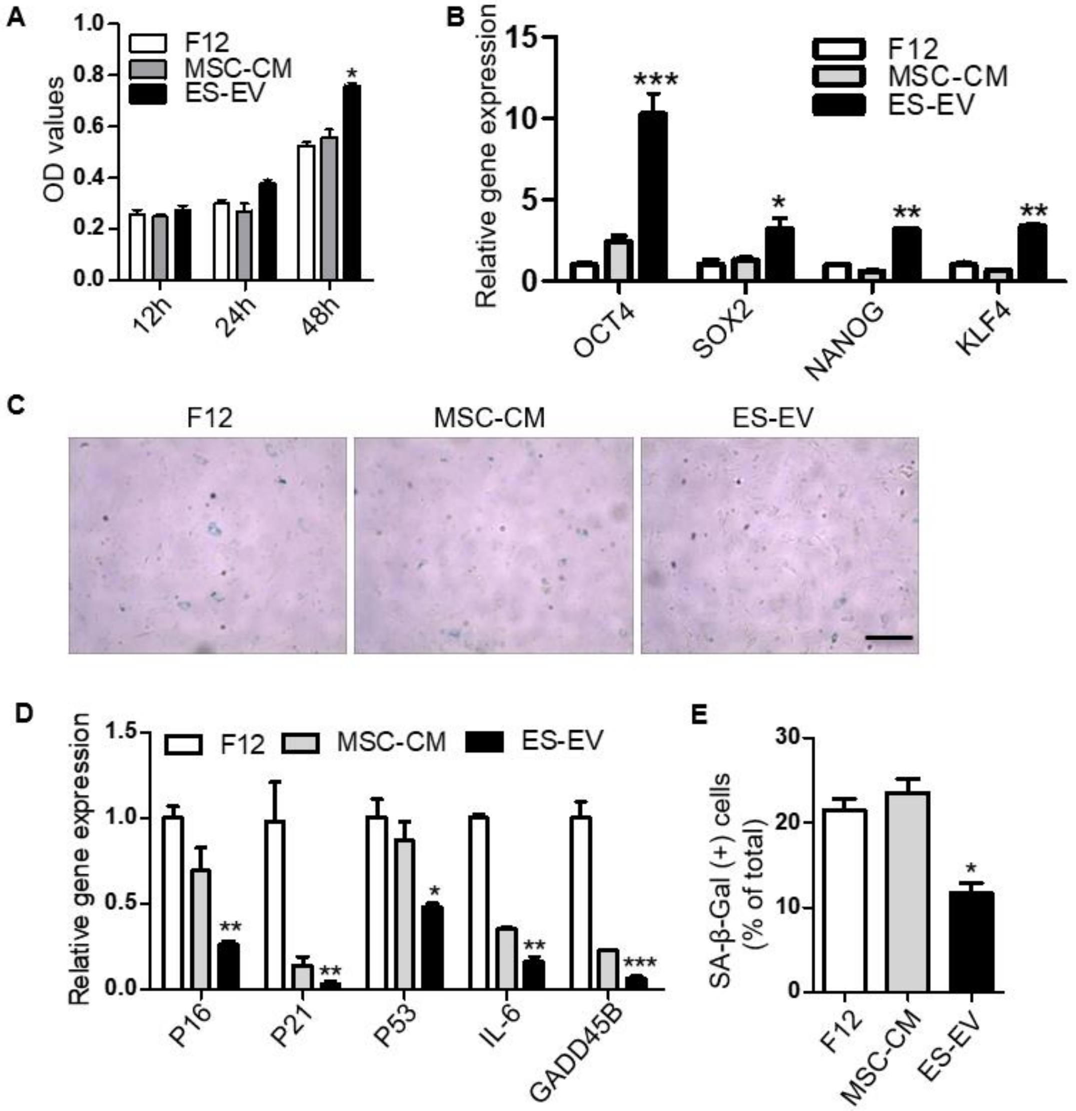

Figure S-4. Antisenescence effects of ES-EV on early-passaged MSCs. (A) The proliferation of early-passaged MSC was measured by MTT. (**B)** qPCR analysis of stemness-related gene expression in early-passaged MSCs. (**C)** SA-β-gal activity staining of early-passaged MSCs treated with F12, MSC-CM, ES-EV for 48h. Scale bar, 100 μm**. (D)** Analyzed of the expression of stress response genes in the p53 pathway, senescence-associated metalloprotease, and interleukin-6 using qPCR. (**E)** Quantification of the percentage of SA-β-gal positive MSCs. Data are presented as the mean ±SEM. (n = 3; *p <.05).

Figure S-5

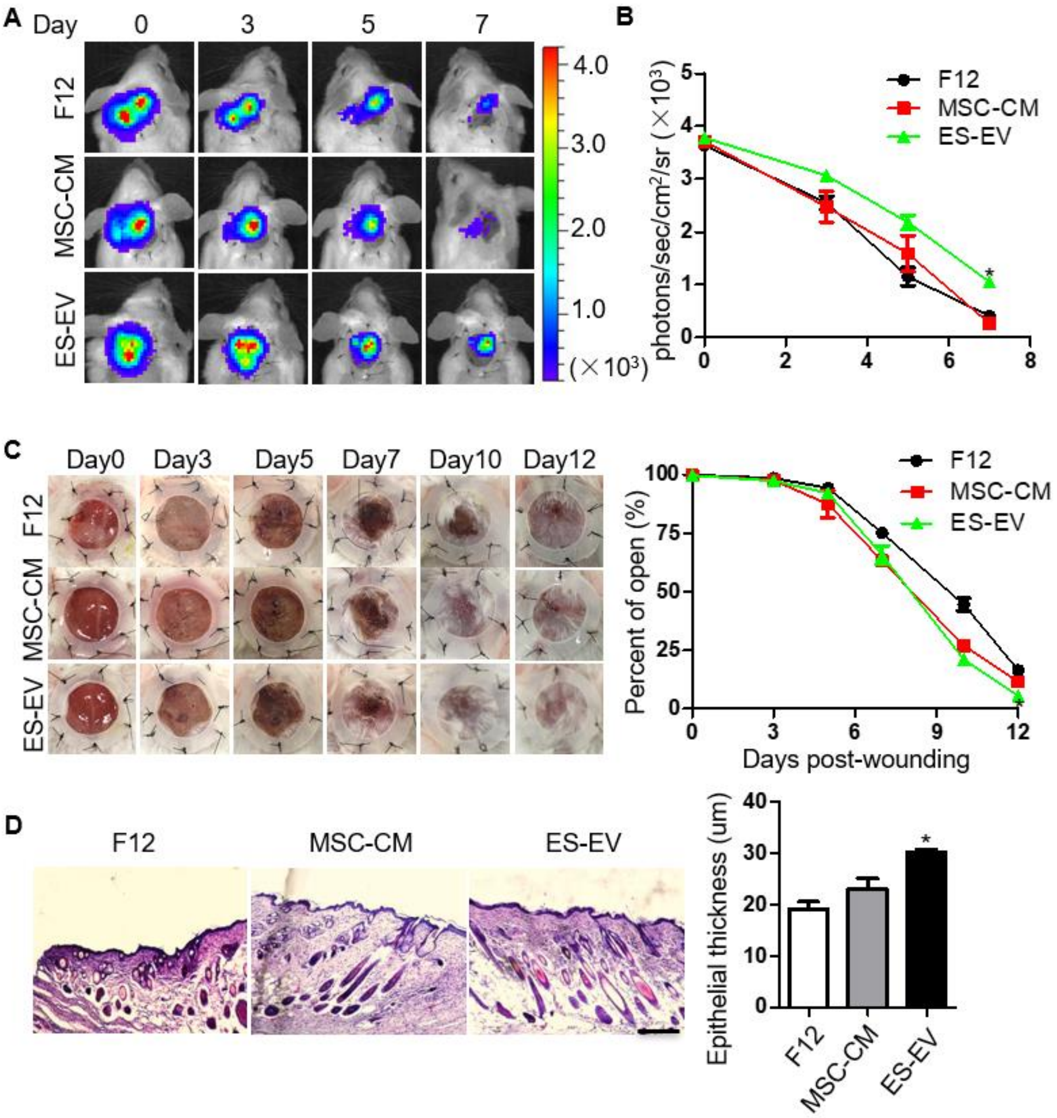

Figure S-5. Enhanced wound-healing process of early-passaged MSCs by ES-EVs. (A) The fate of MSCs after transplantation was tracked by molecular imaging. Images were from representative animals receiving 5×10^5 MSCs with F12, MSC-CM or ES-EVs respectively. (**B)** Quantitative analysis of BLI signals demonstrate that cell survival was improved by ES-EVs at all time points. The ES-EVs group showed significantly better cell survival. Data are expressed as Mean ± SEM. (**C)** Analysis of the wound-healing area within 12 days and the quantitative analysis of wound-healing area. (**D)** Histologic analysis of wound area by HE staining. Scale bar represents 50 μm. Data are presented as the Mean ± SEM. (n = 3; *p <.05).

Figure S-6

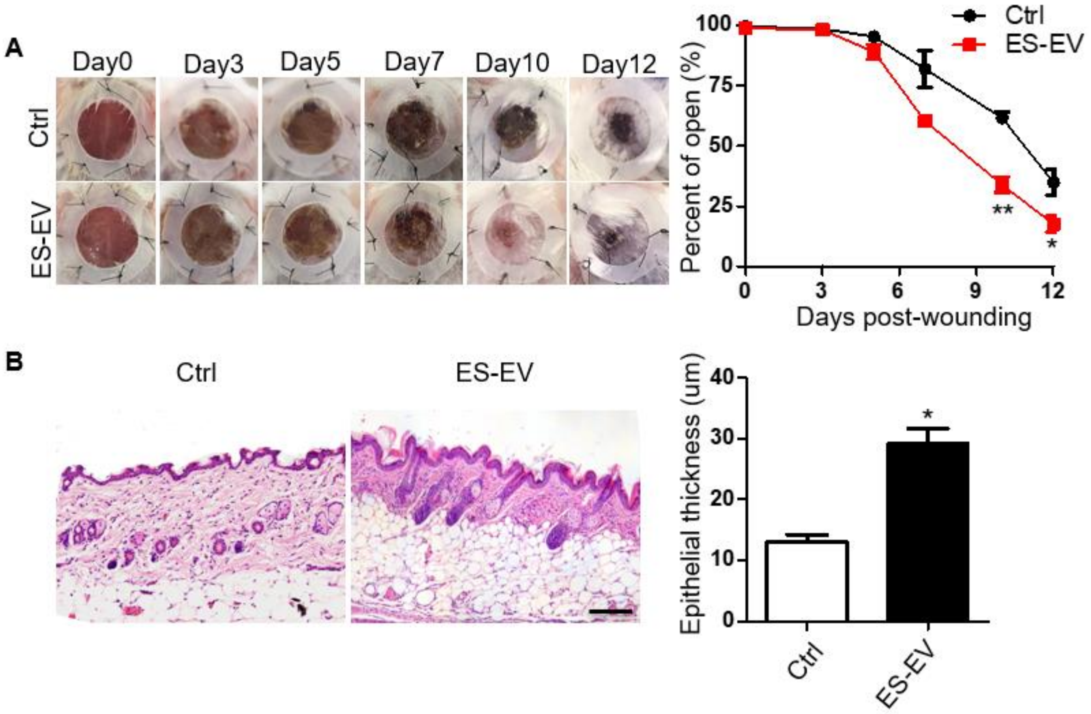

Figure S-6. ES-EVs enhance the regeneration in aged mice. (A) Analysis of the wound-healing area within 12 days and the quantitative analysis of wound-healing area. (**B)** Histologic analysis of wound area by HE staining. Scale bar represents 50 μm. Data are presented as the Mean ± SEM. (n = 3; *p <.05, **p < .01).
